# Occurrence of *Acinetobacter baumannii* genomic resistance islands (AbGRIs) in *Acinetobacter baumannii* strains belonging to global clone 2 obtained from COVID-19 patients

**DOI:** 10.1186/s12866-023-02961-3

**Published:** 2023-08-24

**Authors:** Ghazal Naderi, Mahla Asadian, Pegah Afarinesh Khaki, Mohammadreza Salehi, Alireza Abdollahi, Masoumeh Douraghi

**Affiliations:** 1 Division of Microbiology, Department of Pathobiology, School of Public Health, Tehran University of Medical Sciences, Tehran, Iran; 2 Central Laboratory, Imam Khomeini Hospital Complex, Tehran University of Medical Sciences, Tehran, Iran; 3 Research Center for Antibiotic Stewardship and Antimicrobial Resistance, Department of infectious diseases, Imam Khomeini Hospital Complex, Tehran University of Medical Sciences, Tehran, Iran; 4 Department of Pathology, School of Medicine, Imam Khomeini Hospital Complex, Tehran University of Medical Sciences, Tehran, Iran

**Keywords:** Carbapenem-resistant *Acinetobacter baumannii* (CRAB), COVID-19, Global clone 2

## Abstract

**Aim:**

The *Acinetobacter baumannii* genomic resistance islands (AbGRIs), which were characterized in the genome of the global clone 2 (GC2) *A. baumannii* contain resistance genes. Here, we aimed to determine the occurrence of AbGRIs in GC2 *A. baumannii* obtained from COVID-19 patients in a referral hospital in Tehran, Iran.

**Methods:**

A total of 19 carbapenem-resistant *A. baumannii* (CRAB) isolates belonging to GC2 and sequence type 2 (ST2), including 17 from COVID-19 patients and two from the devices used in the ICU that the COVID-19 patients were admitted, were examined in this study. Antibiotic susceptibility testing was performed by the disk diffusion method. PCR and PCR mapping, followed by sequencing, were performed to characterize the structure of AbGRI resistance islands in the isolates tested.

**Results:**

The AbGRI3 was the most frequent resistance island (RI) detected, present in all the 19 isolates, followed by AbGRI1 (15 isolates; 78.9%) and AbGRI2 (three isolates; 15.8%). Notably, AbGRIs were identified in one of the *A. baumannii* strains, which was isolated from a medical device used in the ICU where COVID-19 patients were admitted. Furthermore, new structures of AbGRI1 and AbGRI3 resistance islands were found in this study, which was the first report of these structures.

**Conclusions:**

The present study provided evidence for the circulation of the GC2 *A. baumannii* strains harboring AbGRI resistance islands in a referral hospital in Tehran, Iran. It was found that resistance to several classes of antibiotics in the isolates collected from COVID-19 patients is associated with the resistance genes located within AbGRIs.

**Supplementary Information:**

The online version contains supplementary material available at 10.1186/s12866-023-02961-3.

## Background

The Coronavirus Disease 2019 (COVID-19) pandemic caused by severe acute respiratory syndrome coronavirus 2 (SARS-CoV-2) is a new health threat in the world [1]. Severe COVID-19 patients require intensive care unit (ICU) admission and usually need mechanical ventilation due to acute respiratory failure [2]. Prolonged mechanical ventilation may lead to an increase in the risk of developing ventilator-associated pneumonia (VAP), especially with multidrug-resistant bacteria such as *Acinetobacter baumannii (A. baumannii)* [3]. *A. baumannii* causes nosocomial outbreaks [4] and is not treatable by most available antibiotics [5]. Most of the extensively drug-resistant (XDR) and pandrug-resistant *A. baumannii* strains (PDR), which have been increasingly reported from different parts of the world, are members of the global clones (GCs) 1 and 2 [6]. The resistance islands (RIs), which contain variable assortments of transposons, integrons, and specific resistance genes, are characterized in the genome of the GCs. The RIs are one of the hallmarks of the horizontal transfer of resistance genes. These include AbaR-type islands (*A. baumannii* resistance islands), and AbGRI-type islands (*A. baumannii* genomic resistance islands) [7-12]. Any or more than one RIs may be carried by MDR *A. baumannii* strains [12]. The AbGRI1 [13], AbGRI2 [10], and AbGRI3 [11] include the major RIs that are identified among GC2 isolates. The AbGRIs include some or all of the genes conferring resistance to antibiotics, including tetracyclines (*tetA*(B), *tetR*(B)) aminoglycosides (*aacC1*, *aacA4, aphA1b*, *aadA1*, *strA*, *strB*, *armA*), sulfonamides (*sul1, sul2*), beta-lactams (*bla*_=*TEM*_), and carbapenems (*oxa23*) [10, 11, 13].

Even though AbGRIs have been identified in GC2 *A. baumannii* strains from various parts of the world [8–12, 14–18], little is known regarding the AbGRIs in Iran. Our previous study demonstrated that the coinfection of *A. baumannii* belonging to GC2 and sequence type 2 (ST2) with SARS-CoV-2 caused outbreaks in a tertiary referral hospital in Iran [19].

This study aimed to characterize the structure of AbGRI1, AbGRI2, and AbGRI3 resistance islands in GC2 isolates collected from COVID-19 patients and also, the GC2 isolates obtained from the medical devices used within the ICU where COVID-19 patients were admitted in the largest referral hospital in Tehran, Iran.

## Results

### Identification of antibiotic resistance profiles

All isolates were resistant to streptomycin, spectinomycin, kanamycin, tobramycin, netilmicin, and cefotaxime. The results of disk diffusion for GC2 *A. baumannii* isolates is presented in Table S 1. In the present study, all *A*. *baumannii* isolates have the MDR phenotype (Table S 1).

### AbGRI1 resistance island

Four out of the 19 GC2 isolates tested (21.1%), did not contain AbGRI1 resistance island (Table 1, not highlighted). In the remaining 15 isolates (78.9%), three groups were distinguished according to their shared characteristics (Table 1, highlighted). The typical AbGRI1 genes, including *strA*, *strB* (conferring resistance to aminoglycosides), *tetA(B)*, and *tetR(B)* (conferring resistance to tetracyclines) were co-located in all isolates. While the interrupted *comM* gene, J2 junction, orf4b adjacent to *comM* gene (indicating that they contained AbGRI1 resistance island), CR2 element, and *oxa23* gene (conferring resistance to carbapenems) were present in all isolates containing AbGRI1, 11 isolates (57.9%) lacked J1 junction, Tn*6022*, Tn*6022Δ1*, and orf region (yellow in Table 1). Furthermore, one of the isolates carrying AbGRI1 lacked the *sul2* gene (conferring resistance to sulfonamides) (blue in Table 1). Long-read sequencing technology such as PacBio or Oxford Nanopore will be required to determine the structure of AbGRI1 in the isolates containing this island.

**Table 1:**
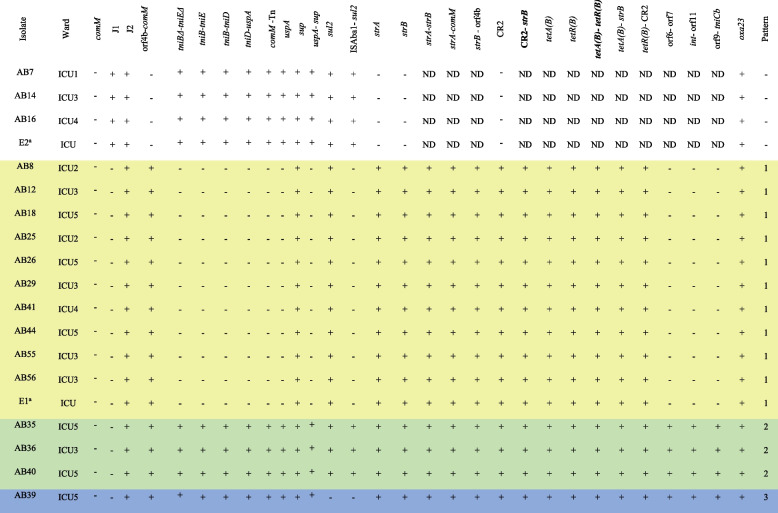
Characteristics of the GC2 isolates containing AbGRI1 resistance island

a. E1 and E2 refer to the GC2 isolates that were obtained from the environment of the ICU where the COVID-19 patients were admitted

Rows highlighted in the same colour indicate the same pattern

PCRs in bold are the linkage PCRs that were performed for identification of AbGRI1s in this study

*ND *Not determined, for the isolates without the orf4b-*comM* fragment, other PCRs were not performed

### AbGRI2 resistance island

Sixteen out of the 19 GC2 isolates tested (84.2%), did not contain AbGRI2 resistance island (Table 2, not highlighted). In the remaining three isolates, two groups were distinguished according to their shared characteristics (Table 2, highlighted). Two isolates (10.5%) contained AbGRI2-12b (yellow in Table 2) and one isolate (5.3%) contained AbGRI2_ABI257_ (an AbGRI2 with a structure that is similar to AbGRI2-12 except for missing a segment from the right-hand side of the island) (green in Table 2). While the *bla*_*TEM*_ (conferring resistance to beta-lactams) was present in all isolates containing AbGRI2, the *aphA1b* (conferring resistance to aminoglycosides) was only present in one isolate carrying AbGRI2 (yellow in Table 2). Furthermore, the *aacC1*, *aadA1* genes (conferring resistance to aminglycosides), and *sul1* genes (conferring resistance to sulfonamides) were not observed in any isolate examined (Table 2).

**Table 2:**
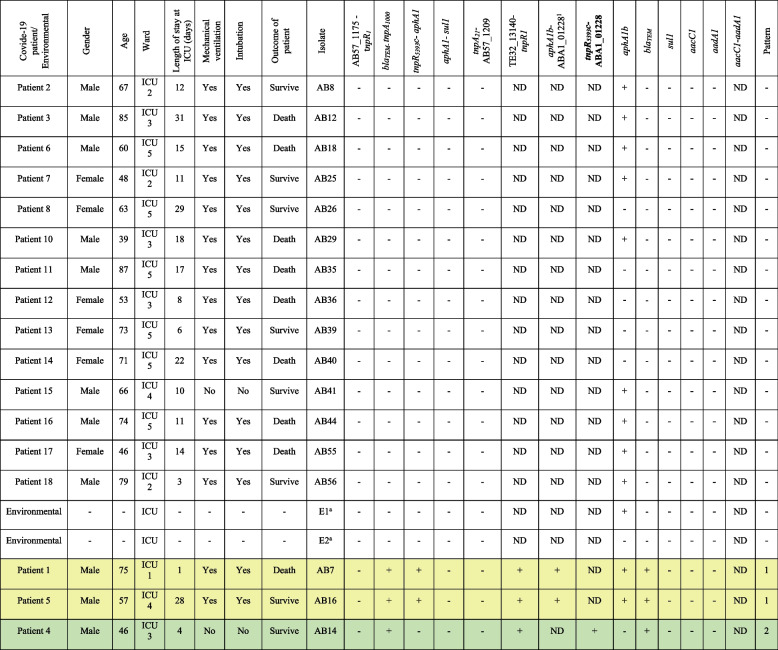
Characteristics of the GC2 isolates containing AbGRI2 resistance island

a. E1 and E2 refers to the GC2 isolates that were obtained from the environment of the ICU where the COVID-19 patients were admitted

Rows highlighted in the same colour indicate the same pattern

The PCR in bold is a new linkage PCR that was performed for identification of AbGRI2 in this study.The predicted size for AbGRI2-12a and AbGRI2-12b is 1581 and 1772 bp, respectively

*ND *Not determined

### AbGRI3 resistance island

It was found that all the isolates carry an AbGRI3 resistance island. Seven groups were distinguished in the isolates according to their shared characteristics (highlighted in Table 3). While the *armA* gene (conferring resistance to aminoglycosides) was present in all isolates, the *aacA4* and *aphA1b* genes (conferring resistance to aminoglycosides) were present in 11 (57.9%) and 12 (63.2%) isolates, respectively (Table 3). AbGRI3-4 was found in seven isolates (36.8%, yellow and green in Table 3); however, a long-read sequencing technique is required to determine the structure of AbGRI3 in the rest of the isolates.

**Table 3:**
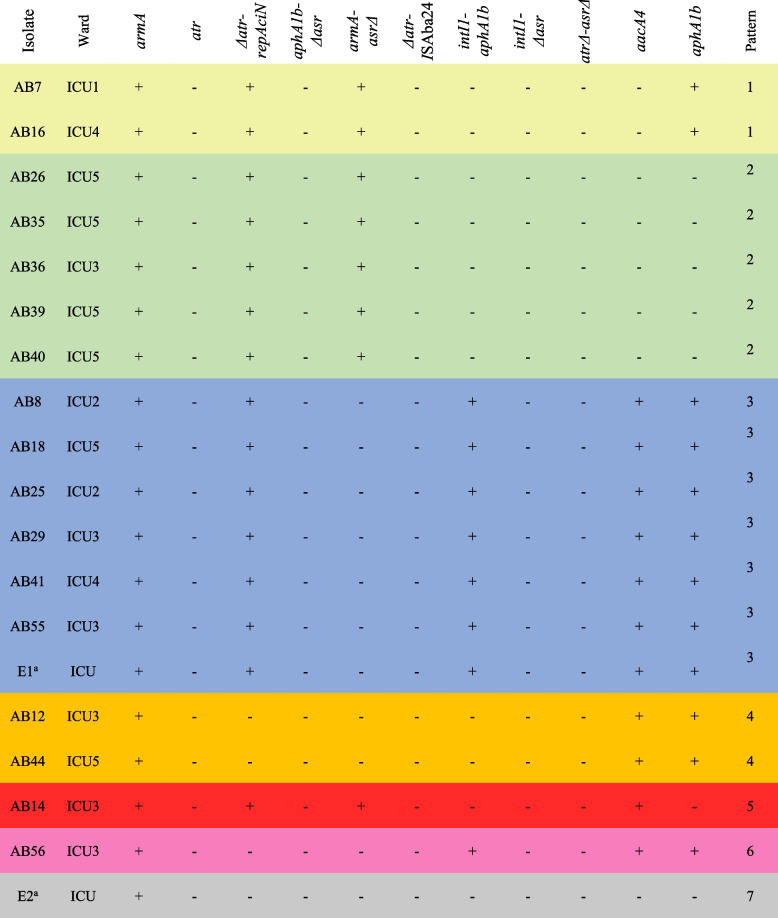
Characteristics of the GC2 isolates containing AbGRI3 resistance island

a. E1 and E2 refers to the GC2 isolates that were obtained from the environment of the ICU where the COVID-19 patients were admitted

Rows highlighted in the same colour indicate the same pattern

## Discussion

Several outbreaks of the CRAB isolates belonging to GC2 in COVID-19 patients in a tertiary referral hospital in Iran were demonstrated in our previous study [19]. Since the isolates exhibited an MDR phenotype, the genetic basis for their phenotype was investigated. In 2013, the term AbGRI was proposed for the RIs that were found in GC2 isolates [10]. The isolates belonged to GC2, therefore, the AbGRI resistance islands carrying resistance genes were studied. Although the features of AbGRI1, AbGRI2, and AbGRI3 resistance islands carrying antibiotic resistance genes were used as informative epidemiological markers to find the relationships between the isolates distributed within the hospital in a study from Iran [20], there was no study to investigate the presence of AbGRIs in GC2 isolates obtained from COVID-19 patients. In the current study, 15 isolates (78.9%) carried an AbGRI1 resistance island. Consistent with our finding, 82.4% of the isolates examined in South Korea [16] and 72.2% of the isolates examined in Iran [20], carried AbGRI1 resistance islands. Here, the isolates that carried AbGRI1 but lacked J1 junction, Tn*6022*, Tn*6022Δ1*, and orf region were found, which is the first report of this structure in *A. baumannii*. In the present study, only two isolates (10.5%) contained AbGRI2-12b, which is consistent with the results of a study by Blackwell, et al. that 15% of GC2 isolates from Singapore carried this island [9]. However, AbGRI2-12b was found in 51.5% and 62.6% of the GC2 isolates investigated in two studies in Iran, respectively [20, 21]. While AbGRI2_ABI257_ with a structure that is similar to AbGRI2-12 but has lost a segment from the right-hand side of the island was found only in one GC2 isolate in this study, it was found in 34% of the isolates in Iran, previously [20]. It was revealed that the *armA* gene was located within the AbGRI3 resistance island in all the isolates containing this gene. Consistent with the results of the current study, Blackwell et al. detected the *armA* gene in 15 out of 20 GC2 *A. baumannii* isolates from Singapore, and they revealed that the *armA* gene is located within AbGRI3 in all the isolates containing this gene [11]. Also, Blackwell et al. found the *armA* in AbGRI3-4 in 46.7% of the GC2 isolates [9], which it is consistent with the presence of AbGRI3-4 in 36.8% of the isolates examined here. However, AbGRI3-4 was found in 80.7% and 77.7% of the GC2 isolates investigated in two studies in Iran, respectively [20, 21]. In addition, five new structures of AbGRI3 resistance islands were found in the current study (patterns three to seven in Table 3), which it is the first report of these structures in *A. baumannii.* This study provided evidence for the circulation of the GC2 *A. baumannii* isolates, which contained at least one AbGRI resistance island, between different ICU wards of a referral hospital. Hence, it is likely that the AbGRIs play a significant role in conferring resistance to various antibiotics in GC2 isolates from Iran. Furthermore, new structures of AbGRI1 and AbGRI3 resistance islands were found in the GC2 isolates obtained from COVID-19 patients in the current study, which were not reported previously. As a limitation, long-read sequencing technology such as PacBio or Oxford Nanopore will be required to determine the structure of AbGRIs in all the isolates containing these islands.

## Conclusions

This study provided evidence that the GC2 *A. baumannii* isolates collected from COVID-19 patients in a referral hospital in Tehran, Iran carry AbGRI resistance islands. It was shown that the MDR phenotype in these isolates is partly associated with the resistance genes located within AbGRIs, including *tetA*(B), *tetR*(B) (tetracyclines), *aacC1*, *aacA4, aphA1b*, *aadA1*, *strA*, *strB*, *armA* (aminoglycosides), *sul1, sul2* (sulfonamides), and *oxa23* (carbapenems).

## Methods

### Bacterial isolates

Among the isolates examined in our previous study [19], 17 CRAB isolates belonging to GC2 and ST2, which were collected from COVID-19 patients, were chosen to examine in this study [19]. In addition, two isolates that were obtained from the medical devices used in the ICU where the COVID-19 patients were admitted (henceforth these isolates referred E1 and E2), were also included in the present study.

### Antibiotic susceptibility testing

In addition to the antibiotics tested by disk diffusion susceptibility testing in our previous study [19], the isolates were also tested using following nine antibiotics (µg per disk) in the present study: streptomycin (25), spectinomycin (25), sulfamethoxazole (300), kanamycin (30), neomycin (30), cefotaxime (30), tobramycin (10), netilmicin (30), and minocycline (30). The results were analyzed according to the Clinical and Laboratory Standards Institute 2023 (CLSI 2023) recommendations for *Acinetobacter* spp [22]. and calibrated dichotomous sensitivity (CDS) (http://cdstest.net/) disk diffusion assay when a CLSI breakpoint for *Acinetobacter* spp. was not available (CDS for streptomycin, spectinomycin, kanamycin, neomycin, and netilmicin).

### PCR assays

#### Characterization of the AbGRI1, AbGRI2, and AbGRI3 resistance islands

The genes associated with AbGRI resistance islands, including *strA*, *strB, sul2, tetA(B), tetR(B*), *sul2, oxa23* (AbGRI1); *aacC1*, *aadA1*, *sul1*, *bla*_*TEM*_ (AbGRI2); *armA*, *aacA4* (AbGRI3); and *aphA1b* (AbGRI2, AbGRI3) were detected by PCR using the primer pairs listed in Tables 4, 6 and 5.

**Table 4:** Primer pairs used for mapping of AbGRI1

PCR	Primer	Sequence (5’-3’)	Annealing temperature (° C)	Amplicon length (bp)	Reference
*comM*	RH927 RH928	CAACCCTGTCTTTGCATTTG GCCAGCAAGCTCAGCATAA	59	880	[8]
*comM*–AbGRI1 (J1)	RH927 RH792	CAACCCTGTCTTTGCATTTG TTCGAGCTTGAAAACTGCAC	60	846	[23]
AbGRI1–*comM* (J2)	RH928 RH916	GCCAGCAAGCTCAGCATAA CCCAAATACTGCCATGTTGA	60	796	[23]
orf4b- *comM*	RH594 RH928	GGCGGATTATCAGTTGTTTCA GCCAGCAAGCTCAGCATAA	60	1844	[8]
**Backbone transposon**					
* tniE*-*tniB*	RH910 RH587	GCGATAGTGAACGGATTGAGA TTGCCCATTAAGCACAACAG	60	560^a^	[24]
* tniD*-*tniB*	RH910 RH584	GCGATAGTGAACGGATTGAGA TCAATATGCCTCGCTCCACT	60	2010	[8]
* uspA*- *tniD*	RH583 RH919	TCCTGTCTCTCGTGTAGCAAT TGTCAAAAATTATTGCATGT	60	3577	[8]
*comM*-Tn	RH791 RH909	TGCTGCAATGAGCTGAAAGT GCGATTCAAAATATCGGTCAA	60	3119	[23]
* uspA*	RH919 RH793	TGTCAAAAATTATTGCATGT CCCAAGAGAGCTGATTTTGC	58	632	[23]
* sup*	RH2523 RH2509	CCCACTTTAGGATCAACGCC GTGGTGTAGTCGCTTGTGTG	60	209	[9]
* uspA-sup*	RH793 RH771	CCCAAGAGAGCTGATTTTGC TGTAAAATCTGGTGGTCGTAC	60	3267	[8]
**Resistance region**					
* sul2*	sul2-F sul2-R	GGCAGATGTGATCGACCTCG ATGCCGGGATCAAGGACAAG	60	407	[25]
ISAba1*-sul2*	ISAba1B sul2-R	CATGTAAACCAATGCTCACC ATGCCGGGATCAAGGACAAG	60	1125	[8]
* strA*	strA-F strA-R	CTTGGTGATAACGGCAATTC CCAATCGCAGATAGAAGGC	58	548	[26]
* strB*	strB-F strB-R	ATCGTCAAGGGATTGAAACC GGATCGTAGAACATATTGGC	58	509	[26]
* strA-strB*	strA-F strB-R	CTTGGTGATAACGGCAATTC GGATCGTAGAACATATTGGC	58	1190	[26]
* strA-comM*	strA-R RH928	CCAATCGCAGATAGAAGGC GCCAGCAAGCTCAGCATAA	60	3509	[8]
* strB-*orf4b	strB-R RH599	GGATCGTAGAACATATTGGC ATACTGTTTCAAAAACTGATGAA	60	2620	[8]
* oxa23*	oxa23F oxa23R	GATCGGATTGGAGAACCAGA ATTTCTGACCGCATTTCCAT	60	501	[27]
CR2	LECR2 RECR2	CACTGGCTGGCAATGTCTAG CTTTGGACCGCAGTTGACTC	60	1793	[8]
**CR2** ***- strB***	strB-F RECR2	ATCGTCAAGGGATTGAAACC CTTTGGACCGCAGTTGACTC	60	2962	[8]
* tetA(B)*	tetB-F tetB-R	TTGGTTAGGGGCAAGTTTTG GTAATGGGCCAATAACACCG	60	658	[28]
* tetR(B)*	RH892 RH893	ACAGCGCATTAGAGCTGCTT AGAAGGCTGGCTCTGCACCT	60	528	[8]
***tetA(B)- tetR(B)***	tetB-R RH893	GTAATGGGCCAATAACACCG AGAAGGCTGGCTCTGCACCT	60	1693	[8]
* tetA(B)-strB*	tetB-R strBout	GTAATGGGCCAATAACACCG AGAGGAGCAACGCGATCTAGC	60	4616	[8]
***tetR(B)-*** **CR2**	RH892 LECR2	ACAGCGCATTAGAGCTGCTT CACTGGCTGGCAATGTCTAG	60	2812	[8]
**orf region**					
orf6-orf7	RH1302 RH1303	CAAATCGGGAAGGTTCAAAA CGGGAAAATTACTGCGATTG	60	1573	[8]
* int*-orf11	RH1306 RH1307	GCATACTCATGTGGTTTAAGACTTG TTAATTGCTTCATCATTTGAGC	60	1638	[8]
orf9-*tniCb*	RH597 RH792	TTTGAAGAAATTGAGCATGAGG TTCGAGCTTGAAAACTGCAC	60	1566	[8]

a.Predicted sizes based on Tn*6022Δ1*. For Tn*6022* is 3410 bp

PCRs in bold are the linkage PCRs that were performed for the identification of AbGRI1s in this study

To investigate the structures of AbGRI1 resistance islands, PCR and PCR mapping experiments were performed to determine interrupted *comM* gene; J1 and J2 boundaries of the AbGRI1; orf4b adjacent to *comM* on the right-hand side of AbGRI1; backbone transposon, resistance region, and orf region (Table 4). To investigate the structures of AbGRI2 resistance islands, PCR and PCR mapping experiments were used to determine the arrangement of the segments including AB57_1175-tnpR_1_, *bla*_*TEM−*_ *tnpA*_*1000*,_ *tnpR*_*5393*_ *c-aphA1b, aphA1-sul1, tnpA*_*21*_ *-*AB57_1209, TE32_13140-*tnpR1*, *aphA1b*-ABA1_01228, and *tnpR*_*5393*_ *c-* ABA1_01228 (Table 6). To investigate the structures of AbGRI3 resistance islands, PCR and PCR mapping experiments were used to determine the *armA* gene, interrupted *atr* gene; and determine the arrangement of the segments including *Δatr-repAciN*, *aphA1b- Δasr*, *armA-asrΔ*, *Δatr*-ISAba24, *intI1- aphA1b*, *intI1- Δasr*, and *atrΔ-asrΔ* (Table 5). The identity of PCR amplicons was confirmed by DNA sequencing.

**Table 5:** Primer pairs used for mapping of AbGRI2

PCR	Primer	Sequence (5’-3’)	Annealing temperature (° C)	Amplicon length (bp)	Reference
AB57_1175 - t*npR*_*1*_	RH1315 RH539	AGGAGATCTTCTTGGCAGTCA CCAGCCCTTCCCGATCTGTTG	60	1051	[10]
*bla*_*TEM−*_ *tnpA*_*1000*_	RH605 RH759	TTTCGTGTCGCCCTTATTCC GCCAGCTCATTTACCTTGCCGA	60	2650	[10]
*tnpR*_*5393c*_ *-aphA1b*	RH520 RH880	CATGGCCCAGCGCGATACTTCAG CAACGGGAAACGTCTTGCTC	60	2297	[10]
*aphA1b- sul1*	RH881 RH751	ATTCGTGATTGCGCCTGAG GCGGAACTTCACGCGATC	60	2712	[10]
*tnpA*_*21*_ *-*AB57_1209	RH668 RH1316	CACCAGAACCGCCTGCTCAA CATCTGCCATCCAGTTTGTG	60	1219	[10]
TE32_13140-*tnpR1*	RH1563 RH539	ATAGATCGGCTTCGGACTCA CCAGCCCTTCCCGATCTGTTG	60	1046	[9]
*aphA1b*-ABA1_01228	RH881 RH2008	ATTCGTGATTGCGCCTGAG TGATGACTTCCATTAAAGCCTGT	60	1581^a^	[9]
***tnpR***_***5393c***_ ***-*** **ABA1_01228**	RH520 RH2008	CATGGCCCAGCGCGATACTTCAG TGATGACTTCCATTAAAGCCTGT	60	1500	[9]
*bla*_*TEM*_	RH605 RH606	TTTCGTGTCGCCCTTATTCC CCGGCTCCAGATTTATCAGC	60	690	[8]
*sul1*	HS549 HS550	ACTAAGCTTGCCCCTTCCGC CTAGGCATGATCTAACCCTCG	60	1100	[29]
*aphA1b*	RH880 RH881	CAACGGGAAACGTCTTGCTC ATTCGTGATTGCGCCTGAG	60	454	[30]
*aacC1*	RH935 RH936	GCAGTCGCCCTAAAACAAAG CCCGTATGCCCAACTTTGTA	60	457	[8]
*aadA1*	RH522 RH531	GTGGATGGCGGCCTGAAGCCA GGCAGCGACATCCTTCGGCGC	60	516	[8]
*aacC1-aadA1*	RH935 RH531	GCAGTCGCCCTAAAACAAAG GGCAGCGACATCCTTCGGCGC	60	2708	[8]

a. Predicted size based on AbGRI2-12a. For AbGRI2-12b it is 1,772

PCR in bold is the linkage PCR that were performed for identification of AbGRI1s in this study

**Table 6:** Primer pairs used for mapping of AbGRI3

PCR	Primer	Sequence (5’-3’)	Annealing temperature (° C)	Amplicon length (bp)	Reference
*armA*	RH2012 RH2013	TCCATTCCCTTCTCCTTTCC GGGGGTCTTACTATTCTGCCTA	60	508	[9]
*atr*	RH2001 RH2004	GGAGTTGGTTTTGGTACAGCA AATGTGGTTGGCGGTTTTTA	60	400	[9]
*Δatr-repAciN*	RH2001 RH2002	GGAGTTGGTTTTGGTACAGCA TATAAGCCACCTCGCTCACC	60	1323	[11]
*aphA1b-Δasr*	RH831 RH2005	TATACCCATATAAATCAGCATCC CACTGATCTGCTGGCTTTCA	60	1203	[11]
*armA-asrΔ*	RH2012 RH2014	TCCATTCCCTTCTCCTTTCC CCAAATACCGCCCACTCAAC	60	1934	[11]
*Δatr-*ISAba24	RH2001 RH2010	GGAGTTGGTTTTGGTACAGCA TTTCGTGACACTCTCGCTTG	60	1605	[11]
*intI1-aphA1b*	RH2003 RH880	GCCTTGATGTTACCCGAGAG CAACGGGAAACGTCTTGCTC	60	1943	[11]
*intI1-Δasr*	RH2003 RH2005	GCCTTGATGTTACCCGAGAG CACTGATCTGCTGGCTTTCA	60	1193	[11]
*atrΔ-asrΔ*	RH2015 RH2006	CCCAGCAATCCATTCGTAGT TGACGAGCTTTGTTTAGGTGTG	60	1524	[11]
*aacA4*	RH532 RH533	GTTAGGCATCACAAAGTACAGC CATCTGGGGTGGTTACGGTACC	60	518	[33]

### Supplementary Information

**Additional file 1.**

## Data Availability

All data generated or analyzed during this study are included in this published article and its supplementary information files. The partial sequences of the Junction 1 (J1), *tetR(B)*-CR2, AB57_1175-*tnpR*_1_, *bla*_*TEM−*_ *tnpA*_*1000*_, *tnpR*_*5393c*_ *-aphA1b, tnpA*_*21*_ *-*AB57_1209, *aacA4*, and *armA* gene of AbGRI3 have been deposited in the GenBank under the following accession numbers: MW092766, OP293342, OM801571, ON240823, ON871819, OP019034 (BankIt2602410), OP650111 (BankIt2625646), and ON982224 (BankIt2602149) (http://www.ncbi.nlm.nih.gov/nuccore/).

## Abbreviations

AbaR: *A. baumannii* resistance island
AbGRI: *A. baumannii* genomic resistance island
CRAB: Carbapenem-resistant *A. baumannii*
XDR: Extensively drug-resistant
GC: Global clone
MDR: Multidrug-resistant
PCR: polymerase chain reaction
PDR: Pandrug-resistant
RI: Resistance island
SARS-CoV-2: Severe acute respiratory syndrome coronavirus 2
VAP: Ventilator-associated pneumonia
