# Supplementary material for: Occurrence of Acinetobacter baumannii genomic resistance islands (AbGRIs) in Acinetobacter baumannii strains belonging to global clone 2 obtained from COVID-19 patients
Source: BMC Microbiol. 2023 Aug 24;23:234. doi: 10.1186/s12866-023-02961-3 (PMC10464229; doi:10.1186/s12866-023-02961-3)
Supplement: Supplementary file 1 — Additional file 1. [file 12866_2023_2961_MOESM1_ESM.docx]

**Table S1**^*^. Results of disk diffusion for GC2 *A. baumannii* isolates collected from the hospital.

| **Isolate** | **Sm** | **Sp** | **Su** | **Km** | **Nm** | **Tm** | **Ne** | **CTX** | **MN** |
| --- | --- | --- | --- | --- | --- | --- | --- | --- | --- |
| AB7 | 6 | 6 | 6 | 6 | 6 | 6 | 6 | 6 | 16 |
| AB8 | 6 | 6 | 6 | 6 | 6 | 6 | 6 | 6 | 8 |
| AB12 | 6 | 6 | 6 | 6 | 6 | 6 | 6 | 6 | 17 |
| AB14 | 10 | 10 | 6 | 6 | 20 | 6 | 6 | 6 | 10 |
| AB16 | 13 | 6 | 6 | 6 | 6 | 6 | 6 | 6 | 11 |
| AB18 | 6 | 6 | 6 | 6 | 6 | 6 | 6 | 6 | 16 |
| AB25 | 6 | 6 | 6 | 6 | 6 | 6 | 6 | 6 | 10 |
| AB26 | 6 | 6 | 6 | 6 | 20 | 6 | 6 | 6 | 11 |
| AB29 | 6 | 6 | 6 | 6 | 6 | 6 | 6 | 6 | 16 |
| AB35 | 6 | 6 | 6 | 6 | 6 | 6 | 6 | 6 | 10 |
| AB36 | 6 | 6 | 6 | 6 | 6 | 6 | 6 | 6 | 17 |
| AB39 | 6 | 6 | 20 | 6 | 21 | 6 | 6 | 6 | 11 |
| AB40 | 6 | 6 | 6 | 6 | 6 | 6 | 6 | 6 | 16 |
| AB41 | 6 | 6 | 6 | 6 | 6 | 6 | 6 | 6 | 10 |
| AB44 | 6 | 6 | 6 | 6 | 6 | 6 | 6 | 6 | 17 |
| AB55 | 6 | 6 | 6 | 6 | 6 | 6 | 6 | 6 | 11 |
| AB56 | 6 | 6 | 6 | 6 | 6 | 6 | 6 | 6 | 9 |
| E1^a^ | 6 | 6 | 6 | 6 | 6 | 6 | 6 | 6 | 16 |
| E2^a^ | 6 | 6 | 6 | 6 | 21 | 6 | 6 | 6 | 11 |

a. E1 and E2 refers to the GC2 isolates that were obtained from the environment of the ICU where the COVID-19 patients were admitted.

^*^Sm: Streptomycin, Sp: Spectinomycin, Su: Sulfamethoxazole, Km: Kanamycin, Nm: Neomycin, CTX: Cefotaxime, Tm: Tobramycin, Ne: Netilmicin, MN: Minocycline**.**

Inhibition zone diameters highlighted white, light gray and dark gray indicate susceptibility, intermediate susceptibility, and resistance, respectively.
